# “There is ‘Plenty’ of Grace—it is Not a Limited Commodity!:” Experiences of Grace in Australian Faith Communities During the Pandemic

**DOI:** 10.1007/s11089-022-01024-0

**Published:** 2022-08-12

**Authors:** Brendan Hyde, Dawn Joseph

**Affiliations:** School of Education, Deakin University, Melbourne Burwood Campus, 221 Burwood Highway, 3125 Burwood, VIC Australia

**Keywords:** Grace, Faith, Resilience, Faith community, Hope, COVID-19, Thematic analysis

## Abstract

During the pandemic years of 2020 and 2021, Melbourne in Australia endured one of the longest lockdowns in the world. Although the severe restrictions for faith communities in particular posed many setbacks, new opportunities for worship were experienced. This paper focuses on a research project that explored hope, grace, and resilience during COVID-19 in Melbourne. A total of 106 participants from a variety of Christian denominations in Melbourne completed an online survey in relation to the notion of grace. Thematic analysis of the qualitative data was employed to explore the lived experiences of the participants. Two overarching themes—God’s grace offers favour, and God’s grace provides strength and builds resilience—are discussed. The findings indicate that while grace is bountiful through faith, it can also be found in unexpected places within faith settings and the wider community. While generalizations from this study cannot be made to other faith communities, recommendations are offered in relation to ways in which ‘church’ may be experienced in 2022 and beyond. The study showed that “there is ‘plenty’ of grace”. Its transformational power offers hope and builds resilience as God’s grace “is not a limited commodity!”

## Introduction

Since the coronavirus hit the global stage in 2020, it has impacted all spheres of life and work, including religious life and practices as well as religious communities (Sulkowski & Ignatowski, 2020). As COVID-19 affected living heritages in varied ways, it simultaneously provided “a source of resilience, solidarity and inspiration for many communities during these difficult times” (UNESCO, 2022). Faith communities sought answers about the pandemic, some believing that “the pandemic is not merely extraordinary but apocalyptic” (Dein et al., 2020, p. 1). Many faith communities, such as Christian congregations, had to adapt their practice and behaviours, some constraining access and others accelerating social transmission to build faith and hope during this unprecedented time (Wildman et al., 2020). Some churches offered live streaming, which enabled them to feel connected in a church setting when congregants were unable to gather in person, where the “practice of spiritual communion provides an opportunity to seek the sustaining grace of God and maintain a connection to the wider Christian community” (Parish, 2020, p. 7).

Many churches used technologies to reach out to their membership due to the varied restrictions and lockdowns that were imposed (Bryson et al., 2020; Hutchings, 2007). While social distancing has become the norm, the notion of physical distancing can impede social connectedness because it assumes disconnectedness (Mansouri, 2020). We conducted our research in Melbourne (Australia), where we work as academics and belong to different Christian faith communities. In 2020 and 2021, Melbourne underwent the longest lockdown with the most severe restrictions in the world during which residence spent a cumulative 262 days under stay-at-home orders (Jose, 2021; Reuters, 2021). Despite these setbacks, the current COVID-19 situation has offered hope for new ways of thinking about faith, hope, and God’s grace.

This paper focuses on a research project that explored hope, grace, and resilience during COVID-19 in Australia. We present survey data from different Christian denominations in Melbourne focused on the notion of grace and collected between November 2021 and mid-May 2022 (*N* = 106). We investigate participants’ perceptions in response to two survey questions:

- What is your understanding of grace?
- In what ways have you experienced God’s grace during the pandemic?

Researchers in the psychology of religion have begun to pay attention to the notion of grace (Bufford et al., 2017; Emmons et al., 2017; Judd et al., 2020), but few have explored the ways in which members of Christian faith communities understood and experienced grace during the pandemic and, more specifically, during the extended lockdowns and restrictions that occurred in various parts of the world, particularly Melbourne. Our study is significant in that it adds to the growing research about ways in which pastoral practice and care contributed to resilience as the pandemic unfolded. Our study centerd on perspectives of faith, grace, and resilience.

### Faith

The notion of faith for Christians is central to trusting in God’s existence and goodness, believing in the elements of various creeds that what God says in God’s Word is true (Buchak et al., 2017). Having a sense of faith gives one hope that God cares, especially when one goes through uncertain and challenging times (Edey & Jevne, 2003). Faith becomes a cornerstone that “protects one from the clouds and storms of hopelessness and despair, providing the ability to see the rainbow of hope for tomorrow” (Darrell, 2016, p. 204). Faith in the postmodern era is rooted in “a common acceptance that beliefs and practices are inextricably embedded in social contexts and personal interests. Because beliefs and practices are never ‘objective’ and therefore ‘pure’, it is not possible to hold unquestionable beliefs” (Martin, 2001, p. 248). For some, faith is a belief in visible outcomes and reality that perhaps aligns with a ‘see-touch culture’ (Martin, 2001). This is more prevalent in the digital era we now live in because messages of faith can be shared through digital media, which can “contribute to personal faith formation, but also puts a mission imperative upon the relationships that Christians [share] online” (Lewis, 2018, p. 529).

According to Scripture, “Now faith is the assurance of things hoped for, the conviction of things not seen” (Hebrews 11:1 NRSVue). While faith may be deemed “a restless thing”, it is about our relationship to God in Jesus (Berkhof, 1920, p. 28) and not an “an ethical system, a set of commandments, nor even a set of doctrines” (McGrath, 2019, p. 1). In light of this, faith comes from believing in God’s Word through God’s son Jesus by hearing or reading the Scriptures. Christians are called to believe and have faith and not to rely on people but on the power of God. Hence, we are saved by grace through faith as we trust in God. Faith can be both passive and active. It is passive when belief in God exists (Bishop, 2016) and active when trust, hope, belief, obedience, and being faithful in Jesus and God’s Word are dynamic (Chia & Juanda, 2020). Believing in God requires making every effort to grow spiritually, which enhances one’s faith, hope, and grace (Coddington, n.d.).

### Grace

Emanating originally from a cluster of Hebrew words—*hesed*, *hanan*, and *raham* and from the Greek word *charis* (Schillebeeckx, 1983)—the word ‘grace’ refers to God’s self-gift to human beings. It is a gift of divine friendship which transforms and heals the lives of people and their experiences, inviting them towards new life (Emmons et al., 2017; Hodge et al., 2020). Further, it is a gift freely given that does not have to be worked for, nor is it dispensed only to those who are deemed worthy of this gift. On this point, theologian Karl Rahner (1968) is clear, indicating that not only is grace freely given but that it is in plentiful supply. For “just because grace is *free* and *unmerited* this does not mean that it is rare (theology has been led astray for too long already by the tacit assumption that grace would no longer be grace if God became too free with it)” (p. 31). God’s unmerited grace, according to Pope Francis (2018, par. 54), is a gift to humankind, freely given and in plentiful supply. Importantly, he maintains that grace is always an act of God’s initiative. This cannot be overstated. It is God who reaches out to humankind with this gift of divinizing loving kindness. As human beings, then, “[W]e cannot buy it [grace] with our works, it can only be a gift born of his [God’s] loving initiative” (Pope Francis, 2018, par. 54).

Although grace is a gift freely given, Berryman (2009) maintains that humankind tends to resist the gift of grace because it can be difficult for people to recognize, either in themselves or in the world around them. For Berryman, grace is nonverbal, and it is present in the attunement between the Creator and creatures who are alive and who generate life. One needs to be mindful of the presence of grace and work with it, even when it is difficult to discern its presence in any given situation. Only when grace is recognized does it have the capacity to profoundly enhance and elevate human flourishing (Emmons et al., 2017). In this way, people experience God’s grace in many powerful ways (Harwood et al., 2021) that build resilience to manage new and challenging life events that impact wellbeing.

### Resilience

Resilience is the ability to recover from or cope with adverse situations (Burnett & Helm, 2013; Eckersley, 2005). Spiritual and religious beliefs may be associated with important resilience resources (Pargament & Cummings, 2010). Qualitative evidence points to engagement of religious coping strategies when facing adversity (Dolcos et al., 2021). Research also verifies a moderate positive correlation between spirituality/religiosity and resilience (e.g., Schwalm et al., 2021). Further, studies have shown the use of spirituality/religiosity as a tool to promote and maintain resilience in life in five key domains, namely reliance on relationships, spiritual transformation, spiritual coping, power of belief, and commitment to spiritual values and practices (Manning et al., 2019).

Resilience is also connected to religious/spiritual wellbeing, where wellbeing is understood to be a person’s overall satisfaction with life (Daykin et al., 2017), impacting the long-term levels of happiness and quality of life (Forgeard et al., 2011). This may result from a self-evaluation of whether one is living a good existence (Villani et al., 2019). Studies have focused on the relationships and connectedness of young people who attend religious organizations as a means of enhancing their spiritual wellbeing, showing that spiritual wellbeing and resilience were found to be interrelated and ecologically bound (Smith et al., 2013). While the resiliency of an individual can be attributed to a belief in the existence of God or the Divine, the wellness indicators of security and satisfaction are directly related to one’s religiosity (Edara et al., 2021).

## Methods

After receiving ethics approval from our institution, we invited Christian faith communities (Catholic, Anglican, Pentecostal, Baptist, Uniting Church, and Evangelical churches) situated in the southeastern suburbs of Melbourne through email to participate in this study. They each received the plain language statement outlining the project. By consenting, they agreed to advertise the project flyer through their church noticeboards, newsletters, and websites. The flyer asked members to voluntarily participate in the survey. It contained a link to the plain language statement and the anonymous Qualtrics survey.

From the 52 faith communities invited, many responded with reasons as to why they were unable to participate. A key reason as to why a number of communities declined the invitation to be involved was their participation in the Australian National Church Life Survey of 2022. They felt it would be imposing on their members to fill out yet another survey. While reminder emails were sent to all invited communities, many did not respond. This may account for the low number of respondents (*N* = 106). The data reported were collected between November 2021 and mid-May 2022.

### Procedure

The Qualtrics survey allowed participants to respond to a range of quantitative and qualitative items (Hagan et al., 2017). The closed questions sought information about the participants’ gender, age, and church affiliation. Questions also included items that sought to understand, in general, the types of activities undertaken during the pandemic (for example, Zoom church, Bible study, gardening and leisure activities) that may have provided participants with a sense of hope and grace. They were able to tick as many listings as possible that applied to them. The responses to these questions provided a snapshot of the broader ways in which participants were able to nurture their faith and relationship with God and others. The open-ended questions probed for deeper responses, for example, How has God sustained you during this time of COVID-19? In what ways did you feel supported by your faith community? What is your understanding of grace? Can you think of an example when you experienced grace during the pandemic? The survey was initially trialed and tested for ambiguity with lay church members, senior and junior clerics, and an overseas and interstate church musician. It was then amended before dissemination.

### Data analysis

Thematic analysis (TA) was employed to explore the lived experiences of our participants (Clarke & Braun, 2017). As a tool of analysis, TA is a method for identifying, analyzing, and interpreting patterns of meaning, or ‘themes’ in qualitative data (Creswell & Creswell, 2018; Nowell et al., 2017). It can be applied across a range of theoretical frameworks and research paradigms, and it emphasizes an organic approach to coding and theme development (Clarke & Braun, 2017). A key feature of TA is its flexibility, not just its theoretical flexibility but also its flexibility in terms of the research question, sample size and constitution, data collection method, and approaches to generating meaning (Clarke & Braun, 2017). As we were interested in participants’ experiences and/or understandings of grace, TA enabled us to understand what the participants thought, felt, and did in relation to their understandings and experiences of grace. Independently, we each read and re-read the data provided by our participants to identify emerging patterns and themes. These were then grouped into broad overarching themes (Nowell et al., 2017). We use direct quotations from the participants to illustrate our findings (Flick, 2002; Kelley et al., 2003) and discuss the two overaching themes (see Table 1).

**Table 1:** Survey Themes Emerging from the initial coding

Initial Coding	Emergent Themes	Overarching Themes
Kindness, benevolence, favour	Grace is a gift	God’s grace offers favour
Enables us to be more than we can be		
Gentleness		
Good will	Grace is an unexpected, undeserved blessing and favour	
Free gift from God		
God’s life, God’s love		
God’s blessing		
Freely given		
Undeserved		
Confidence	Grace is experienced in shared moments of service to others	God’s grace provides strength and builds resilience
Small acts		
Shared moments		
Helping others		
Strength and motivation		
Resilience	Grace provides strength and resilience	
Sustaining		

## Results

Of the 106 respondants, 48% were Anglican, 14% Uniting Church, 11% Catholic, 9% Pentecostal, 4% Evangelical, and 2% Baptist, with 12% identifying as ‘other’. 72% of participants were female, 26% were male, and 2% identified as ‘other’. All participants were over the age of 18, with 16% aged between 50 and 59, 10% between 60 and 69, 9% between 70 and 79, and 10% between 80 and 89. The largest number of participants comprised the age group 30 to 39 (19%), with 8% aged between 40 and 49 and 3% over the age of 90. There were also 8% aged between 18 and 29. 17% of participants did not provide their age. In this section, we report on the findings in relation to the themes of God’s grace offering favour and freedom and God’s grace providing strength and building resilience.

### God’s grace offers favour

A number of the survey respondents indicated grace was a “gift” from God “freely given”. The notion of grace enables participants to be more than who they think they can be. For instance, participants said that grace is:

- when God’s kindness, benevolence and favour is bestowed on you.
- offering and receiving kindness, gentleness, and goodwill.
- growing in God’s love where we find forgiveness for ourselves and others.
- where we find peace.
- a gift from God with no strings attached—we are forgiven.
- more practical than theoretical—it is an action word. It is doing practical things in people’s lives.

Additionally, a number of participants indicated that grace was “God’s self-gift to humankind” and that this gift of grace was “free and unmerited”. They stated, for instance, that:

- grace is a part of God’s character.
- [grace] is God’s blessing for all those who wish to accept it and is a beautiful thing.
- grace is God’s life and love active within each of us.
- grace is His life and power given to us by His favour.
- we all have grace, even if we feel that we don’t deserve it. Grace is shown in the ways that we act towards others. There is ‘plenty’ of grace—it is not a limited commodity!
- grace is the free gift of God, often the unexpected blessings.
- grace is God’s unmerited favour.
- grace keeps us grounded and able to function as channels of God’s healing, life-giving love, even in difficult circumstances.

One participant astutely and succinctly summed these ideas up by saying:

Grace is something you can’t buy; it is God’s love and sanctification towards us. It is his favour upon us as he daily forgives and restores us unto himself. It is something we need from God to live a new life. Grace is God’s mercy upon us, it is God’s character.

### God’s grace provides strength and builds resilience

Participants spoke about their experiences of grace during the pandemic and how it might have sustained them in their faith, noting that these experiences often occurred in shared moments of service to others. They indicated that grace “gave us the confidence to carry on as many of our normal activities as possible” and that the “small acts that I saw people make toward each other renewed my sense of faith, not just in people, but just my sense of faith”. One participant stated that she experienced grace “when I cared for my Mum during her illness”, while another indicated that grace was experienced when “our parish was able to provide emergency food and supplies for another parish who had suffered during terrible storms,” and that “even in lockdown our parishioners would drop off food at the vicarage doorstep for this cause. Six carloads in total!” Another participant said grace was experienced when “people connected and displayed more tolerance than I’d seen before”. One participant summed this up well by saying:

I experienced grace when I saw images of the medical teams on the television helping people who had been admitted with COVID-19, even though they were putting themselves at risk. I experienced grace when others acted with kindness towards me.

Particpants reported grace contributing positively towards their wellbeing, saying that:

- having grace gives me strength and the belief that I can do anything.
- grace has made me feel more resilient—that I could cope, that I could get through the pandemic.
- grace has given me the strength to continue caring for my clients and families.
- grace keeps me focused and strengthened...making me resilient.
- grace disciplined my thought process and kept me sane.

One participant summed up the way in which grace contributed positively towards her wellbeing:

I believe grace has given me the strength and motivation to get through a very difficult time. I have found an inner strength I never realized I had. It has helped me to move forward and fulfil my promises to my parents to take care of things after their passing.

Similarly, another said:

God’s saving grace and his word has sustained me as I go on my knees and recall Jeremiah 33:3 “Call to me, and I will answer you, and show you great and mighty things, which you do not know.” When I am feeling sad, confused, hurt, lonely, or angry, grace has sustained me. God’s grace sustains me when I need to encourage or motivate others in my family or amongst friends and in my workplace with colleagues.

However, some participants in our study indicated that they did not experience an outpouring of grace during the lockdown. For instance, one particular participant, while not necessarily losing hope, maintained that grace was a rare experience during the pandemic. This participant stated:

It’s hard to think how I got it, and how I felt any grace during the pandemic. I think I was just feeling too distant from God through most of the pandemic. I can think through so many times in my life I have received God’s grace, but during the pandemic—that’s difficult!

Similarly, another participant said that they felt grace was lacking when isolating after having come in contact with a COVID-positive case. This person described “feeling abandoned during a two-week period of home isolation as a close contact of a Positive COVID Case. There was no contact with anyone. There was no experience of grace then!”

Another participant indicated that there was a lack of grace in their enforced diminished church involvement. This participant felt “unnoticed at church” and that her “ministry was ignored” during the pandemic. “I didn’t experience God’s grace then”, she said. Further, this lack of an experience of grace was negatively impacting her wellbeing, resulting in a sense of reduced purposefulness. “I just didn’t feel valued”, she said.

## Discussion

Our findings suggest that members from faith communities kept in touch with each other by using various formats of online technology (livestreaming, Zoom, Facebook, etc.) to communicate and collectively support one another through the anxious time of the pandemic (Bryson et al., 2020). The findings show that people referred to specific Scripture verses to illuminate their experiences and connect with God and were able to share and affirm their faith as a way to dispel the darkness and fears of the unknown through God’s grace (Edey & Jevne, 2003). The discussion below centers on the two overarching themes outlined in Table 1 above.

### God’s grace offers favour

By exercising their faith, many participants found a sanction of God’s grace by standing on God’s word, having a relationship with God (Berkhof, 1920). Believing in God’s mercy and goodness as God’s *charis* (Schillebeeckx, 1983), they received God’s grace and unmerited favour. At a time when the world seemed broken because of the pandemic, their utterance of “God heard my cry” or “God gave me and my family grace” may have come to the fore as people tended to trust God during this time of uncertainty. For most respondents, their faith in God brought solace during hard times, especially when Melbournians endured the longest lockdown in the world (Jose, 2021; Reuters, 2021). They were unable to meet with family, friends, and fellow churchgoers. For some, being unable to receive Holy Communion in person was a new phenomenon and was potentially disembodied, with “the essence of Christianity [becoming] a cacophony of information and observation and cease[ing] to be a religion that is lived out within the human, physical community” (Parish, 2020, p. 6).

Many respondents felt inspired and invigorated as they identified the many ways in which they had experienced God’s unmerited favour (Emmons et al., 2017). This is summed up by a respondent who said, “We had a sense of peace and felt assured God was caring for us”, while another found “believing in God and knowing he is always around guiding and supporting me” gave them hope in being able to weather the storms of life (Darrell, 2016). Another one strongly felt her form of grace as an act of love, caring for a parent as “the most humbling and selfless thing we can do”.

The findings reveal that people trusted in God’s favour and were able to believe “it is well with my soul.” As many lost their livelihoods, one person remarked, “Grace was freely given through God’s provision in spite of lack of income”. Faith became the substance that brought the realm of the supernatural into the natural as respondents drew on the Scriptures through Bible studies and sermons to walk with God and to trust in God’s unmerited favour as they lived by faith. Not having faith may cause followers to become ineffective in their walk with Christ, which is not pleasing to God. Therefore, spending time with God and the Scriptures, as our participants found, empowered them to remain positive during challenging times. One person found that “every time I experienced things which inwardly would make me upset, I then found the favour of God’s Grace was greater”. It was apparent that God’s grace came through for many respondents in different ways, including God’s favour they felt they did not deserve (Berryman, 2009). Some mentioned it was difficult to feel God’s presence or recognise God’s grace at times when going through hardship of loss of work or income or health. Nonetheless, the word of God promises God’s “grace is sufficient for you” (2 Corinthians 12:9 NRSVue), which may have bolstered their sense of hope during the pandemic.

### God’s grace provides strength and builds resilience

Our findings also suggest that these participants experienced grace in shared moments of service to others and that these shared moments provided strength for them and built resilience. This is, in part, consistent with the literature finding that religious beliefs may be associated with important resilience resources (Pargament & Cummings, 2010) and that religious coping strategies are utilized when people face adversity (Dolcos et al., 2021). What is interesting here, though, is that grace was not experienced as a passive phenomenon. Rather, participants experienced grace through the activity of being of service to others, either in their families or in their faith communities. While grace is experienced through sacraments, prayer and meditation, and the saints (Harwood et al., 2021), our findings reflect a closer understanding of Berryman’s (2009) idea of the presence of grace and of working with it. Berryman gives the example of St. Paul’s chapel in New York on 11 September 2001 as a place in which grace was experienced in the activity of service, when “Holy communion was celebrated amidst people who were eating, coming and going, massaging aching bodies, treating sore and injured feet, murmuring words of counsel and care, and sleeping in the pews with teddy bears to soften the tragedy and tears” (p. 215). In a similar way, the participants in our study experienced grace when they “provided emergency food and supplies for other parishes who had suffered during terrible storms” during the pandemic in Melbourne. They also mentioned the verb “doing” as a way to experience grace. One summed up this by saying it is “in the small acts of love towards other people we experience grace”.

The various acts of grace experienced by the participants offered them strength, building their resilience. The acts of service within family and the community may have offered participants a greater sense of satisfaction, happiness, and wellbeing during hard times (Daykin et al., 2017; Forgeard et al., 2011). Despite the difficulties, participants were able to gauge for themselves whether or not they were living fulfilling lives (Villani et al., 2019). They intimated that grace may have made them feel more resilient and that it had given them the strength and motivation to endure day-to-day living. Many spoke of drawing on the Scriptures or online services to sustain their faith and reach out to others. One summed it up by saying, “I found an inner strength that I did not realize I had as I relied more on God”; another found during this time that she had “greater empathy towards others as their feelings and experiencing were not always known”.

The findings show people seemed to be supporting each other more during the pandemic. Since Melbournians were in lockdown and experienced high restrictions, they connected with each other using social media and technology as a way to support one another and nurture new and old relationships (Manning et al., 2019). One respondent summed this aspect up, saying, “A friend calls me just about every day to chat/check on me despite the trials and tribulations she is going through at her home and work”. In this way people, reached out as an act of grace, as this respondent said: “I am not forgotten and the promise to call is constant. Though I am not always in the best frame of mind, my friend always calls. She shows love, care, and support. Now, that’s Grace!”

However, not all participants experienced grace as providing strength and building resilience. For some, while God’s grace had been experienced throughout life generally, it somehow seemed distant during the pandemic. This may be reflective of St. John of the Cross’s (1953/2017) notion of the dark night of the soul, which explains, by means of a narrative journey, the painful experiences that people endure as they seek to grow in spiritual maturity and union with God. That some participants may have experienced the dark night of the soul during the pandemic does not imply that they lack faith or that they are not resilient. The poet Tennyson (1850) astutely and famously noted, “There lives more faith in honest doubt, / Believe me, than in half the creeds” (In Memoriam A.H.H. OBIIT MDCCCXXXIII: 96). Rather, it suggests that our findings may not always reflect the notion of resilience but rather of vulnerability—of being exposed and opened to the ill effects of the pandemic on social life and connectedness. Importantly, Huta and Hawley (2010) note that strengths and vulnerability sometimes interact, with strengths weakening the relationship between vulnerability and wellbeing. Thus, vulnerability can be an important and sometimes overlooked contributor to a person’s sense of wellbeing.

It may also be the case that some participants, despite the anonymity of the Qualtrics survey instrument, may have felt the need in their responses to say ‘all of the right things’, thereby presenting a positive outlook on their experiences of grace. Thus, even though they may have experienced moments of vulnerability, and perhaps times when grace may have seemed distant during the pandemic, they nonetheless provided responses that they believed the researchers might have wanted to hear—responses that the researchers were looking for. Thus, it is possible that a greater number of participants in our study than is reflected in the data may not have always experienced grace as providing strength and building resilience.

## Conclusions

The findings from this study largely demonstrate that grace is bountiful and can be found in unexpected places. Despite the cumulative effect of two years of the pandemic wearing on people in different ways, our participants discovered grace through prayer, the Scriptures, and online church services. They placed their trust and hope in God by having faith that God would show mercy and answer their prayers. The unmerited favour of grace was experienced in active and practical ways. It provided strength, built resilience, and increased their faith. While our participants said they were challenged in various aspects of life during this time, it would seem that it was “by God’s grace or through his grace [they were] able to keep strong in the face of Christ, and [were] prayerful for all that lies ahead” (Freire, 2022, p. 2).

We recognize that there are limitations to our study, including the small sample size and the restriction of our project to Christian faith communities in Melbourne, which experienced one of the longest lockdown periods in the world between 2020 and 2021 (Jose, 2021; Reuters, 2021). Therefore, statistical inferences and generalizations to Christian churches outside Melbourne and in other parts of the world cannot be made. Investigating the responses from people in larger and more varied faith communities where there might be an array of different theologies and belief systems may yield a more robust set of findings.

From our modest set of findings, we recommend that members of faith communities be encouraged to share their experiences of prayer, hope, and grace with others, which may increase faith and build a stronger relationship with God and the wider community. Additionally, members should be given the opportunity to offer their gifts and expertise to contribute to outreach programs (faith-based and secular) where faith, hope, and grace help build resilience. In light of the pandemic, blended modes of worship have forced people to congregate in new and different ways. Therefore, we recommend that church leaders consider different ways in which ‘church’ might be experienced in 2022 and beyond in relation to the integration of new technologies, different ways of gathering to worship, and sharing of resources. Since “there is ‘plenty’ of grace,” its transformational power offers hope, which may revive faith, rekindle charity, and build resilient communities, as God’s grace “is not a limited commodity!”

As a result of this particular study, we anticipate a larger research project that will explore an array of faith communities where there may be varied theologies and belief systems in the State of Victoria, of which Melbourne is the capital city. This may yield a more complete understanding of participants’ understanding of grace and how they experienced grace during the pandemic.
